# Extracellular α-synuclein induces sphingosine 1-phosphate receptor subtype 1 uncoupled from inhibitory G-protein leaving β-arrestin signal intact

**DOI:** 10.1038/srep44248

**Published:** 2017-03-16

**Authors:** Lifang Zhang, Taro Okada, Shaymaa Mohamed Mohamed Badawy, Chihoko Hirai, Taketoshi Kajimoto, Shun-ichi Nakamura

**Affiliations:** 1 Division of Biochemistry, Department of Biochemistry and Molecular Biology, Kobe University Graduate School of Medicine, Kobe 650-0017, Japan

## Abstract

Parkinson’s disease (PD) is the second most common neurodegenerative disorder. The presence of α-synuclein (α-Syn)-positive intracytoplasmic inclusions, known as Lewy bodies, is the cytopathological hallmark of PD. Increasing bodies of evidence suggest that cell-to-cell transmission of α-Syn plays a role in the progression of PD. Although extracellular α-Syn is known to cause abnormal cell motility, the precise mechanism remains elusive. Here we show that impairment of platelet-derived growth factor-induced cell motility caused by extracellular α-Syn is mainly attributed to selective inhibition of sphingosine 1-phosphate (S1P) signalling. Treatment of human neuroblastoma cells with recombinant α-Syn caused S1P type 1 (S1P_1_) receptor-selective uncoupling from inhibitory G-protein (Gi) as determined by both functional and fluorescence resonance energy transfer (FRET)-based structural analyses. By contrast, α-Syn caused little or no effect on S1P_2_ receptor-mediated signalling. Both wild-type and α-Syn(A53T), a mutant found in familiar PD, caused uncoupling of S1P_1_ receptor, although α-Syn(A53T) showed stronger potency in uncoupling. Moreover, S1P_1_ receptor-mediated β-arrestin signal was unaltered by α-Syn(A53T). These results suggest that exogenous α-Syn modulates S1P_1_ receptor-mediated signalling from both Gi and β-arrestin signals into β-arrestin-biased signal. These findings uncovered a novel function of exogenous α-Syn in the cells.

Parkinson’s disease (PD) is the second most common progressive neurodegenerative disorder after Alzheimer’s disease. The pathological hallmarks of PD are selective loss of the dopaminergic neurons in the substantia nigra pars compacta and the presence of α-synuclein (α-Syn)-positive intracytoplasmic inclusions, known as Lewy bodies, which develop in the cell body of affected neurons in both idiopathic^1^,^2^,^3^ and hereditary PD, i.e., missense mutations, α-Syn(A53T)^4^, α-Syn(A30P)^5^, and α-Syn(E46K)^6^ as well as multiplication in the α-Syn gene^7^,^8^.

α-Syn with 140 amino acids is highly expressed in neurons and enriched in synaptic terminals suggesting a role in synaptic function and plasticity^9^,^10^. α-Syn is a natively unfolded molecule that can self-aggregate to form oligomers and fibrillar intermediates^11^,^12^. Subsequently, it has been suggested that oligomers rather than fibrillar structures might actually show toxicity to cells through binding to membrane lipids and causing membrane perturbations^13^,^14^. It has also been suggested that α-Syn has an ability to interact with gangliosides in the cholesterol and sphingolipid-rich membrane microdomains known as lipid rafts, and that it has a potency to alter the functions of several signalling molecules at the raft regions^15^. Recent studies suggest that α-Syn is detected in cerebrospinal fluid and plasma^16^,^17^, and cell-to-cell transmission of α-Syn plays a role in the progression of PD, *i.e.*, α-Syn pathology is initiated in the peripheral nervous system and olfactory bulb, ascends toward the brainstem and into the midbrain such as substantia nigra, and then eventually spreads to the forebrain as suggested by “Braak’s hypothesis”^18^. It remains elusive as to how extracellular α-Syn participates in the pathogenesis of PD.

Our laboratory has recently demonstrated that extracellular α-Syn causes inhibition of platelet-derived growth factor (PDGF)-induced chemotaxis in human neuroblastoma-derived SH-SY5Y cells through selective suppression of rac1 activation which is required for cell migration^19^. It is known that the ability of growth factors such as PDGF to enhance cell migration utilises the transactivation of sphingosine 1-phosphate (S1P) receptors in a variety of cell types^20^. S1P is a phosphorylated product of sphingosine catalysed by sphingosine kinase (SphK) and has been implicated in an important lipid mediator acting both inside and outside the cells^21^,^22^. S1P binds to members of GTP-binding protein (G-protein)-coupled S1P receptor family (S1P_1–5_) and triggers diverse cellular processes, including cell angiogenesis, cardiac development, immunity, cell motility, neurotransmitter release and endosome maturation^23^,^24^,^25^,^26^.

From this background we have studied the extracellular effects of α-Syn on S1P signalling-mediated cell motility using both functional and fluorescence resonance energy transfer (FRET)-based structural analyses. Here we show that extracellular α-Syn causes S1P_1_ receptor–selective uncoupling from inhibitory G-protein (Gi) while other signalling events including S1P_2_ receptor-mediated signalling remain intact. A possible interpretation of α-Syn-induced changes in cellular signalling in conjunction with pathophysiological relevance is discussed herein.

## Results

### PDGF utilises transactivation of S1P_1_ receptor for maximal chemotaxis

**Figure 1:**
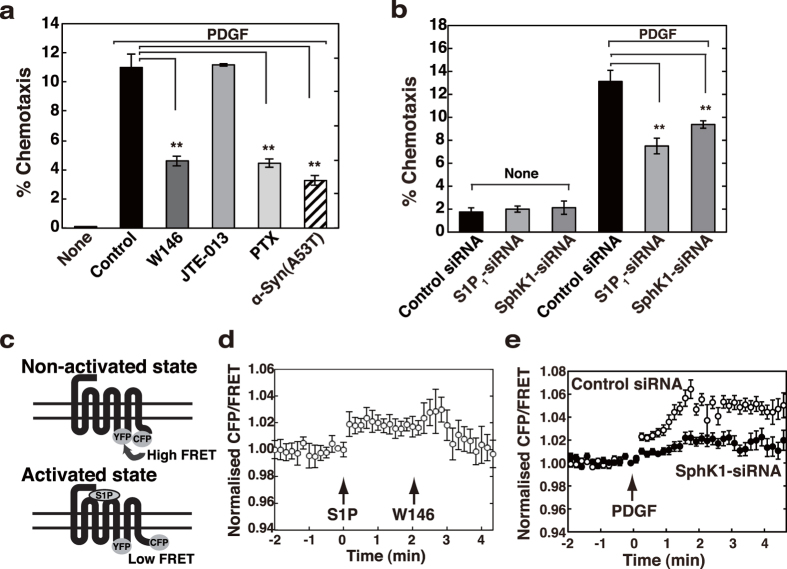
Involvement of S1P signalling in PDGF-induced chemotaxis. (**a**) SH-SY5Y cells were cultured for 18 hr in the upper chamber with vehicle (control), 10 μM W146, 10 μM JTE-013, 100 ng/ml PTX or 1 μM α-Syn(A53T). The cells migrated into the lower chamber in the absence (none) or presence of 20 ng/ml PDGF were counted. PDGF-induced chemotaxis was expressed as % chemotaxis. Values represent means ± s.e.m. of three independent experiments carried out in triplicate. Statistical significance was analysed by Student’s t-test (**P < 0.01). (**b**) SH-SY5Y cells transiently transfected with control, S1P_1_ receptor- or SphK1-siRNA were cultured for 24 hr and then plated on the upper chamber in the absence of serum. The cells migrated into the lower chamber in the absence (none) or presence of 20 ng/ml PDGF were counted. Values represent means ± s.e.m. of 3 independent experiments carried out in triplicate. Statistical significance was analysed by Student’s t-test (**P < 0.01). (**c**) A schematic diagram for a FRET-based probe to detect conformational changes in S1P_1_ receptor was depicted. CFP and YFP were separately fused to the same receptor molecule. Under non-activated conditions these two fluoroprobes associate closely (high FRET), whereas S1P-induced conformational changes of the receptor cause their dissociation (low FRET). (**d**) Cells transiently expressing this FRET probe in (**c**) were serum-starved for 18 hr and stimulated with 100 nM S1P (first arrow) and analysed for FRET in living cells. Two min after S1P stimulation, 10 μM W146 was added (second arrow). (**e**) SH-SY5Y cells cotransfected with control or SphK1-siRNA together with vectors encoding this FRET probe in (**c**) were serum-starved for 18 hr and stimulated with 20 ng/ml PDGF (arrow) and analysed for FRET in living cells. A representative emission ratio of the 2 fluorophores from 5 independent experiments is shown.

It has recently been reported from this laboratory that extracellular α-Syn inhibits PDGF-induced chemotaxis^19^. To identify signalling pathway, which is important in PDGF-induced chemotaxis and that is sensitive to extracellular α-Syn, we first examined the involvement of S1P signalling in this phenomenon. An S1P_1_ receptor-specific blocker, W146, inhibited PDGF-induced chemotaxis to an extent similar to α-Syn(A53T) treatment (Fig. 1a). Since S1P_1_ receptor is known to couple exclusively with Gi^27^, it is reasonable to assume that pertussis toxin (PTX) inhibits the cell motility. In contrast, a selective S1P_2_ receptor antagonist JTE-013 showed no effect on PDGF-induced chemotaxis. Involvement of S1P signalling in PDGF-induced chemotaxis was further confirmed by downregulating one of the subtypes of SphK, SphK1 expression using small interfering RNAs (siRNAs). SphK1-siRNA caused inhibition of PDGF-induced chemotaxis by 30% as compared with the control siRNA (Fig. 1b). Similarly, knockdown of S1P_1_ receptor by S1P_1_ receptor-siRNA caused 40% inhibition of PDGF-induced chemotaxis. These results indicate that S1P signal is involved in PDGF-induced chemotaxis, consistent with a previous report^20^. As described in a previous study showing a successful detection of conformational changes in α2A-adrenergic receptor using a fluorescence resonance energy transfer (FRET)-based technique^28^, we have similarly constructed a probe to detect conformational changes in S1P_1_ receptor using a FRET technique, where the cyan fluorescent protein (CFP) and yellow fluorescent protein (YFP) were separately fused in the same S1P_1_ receptor molecule (Fig. 1c). Under a resting state these fluorophores are closely situated, which enables FRET to occur, whereas the specific agonist (S1P) stimulation causes conformational changes in the receptor, resulting in FRET decrease. Upon stimulation by S1P, cells expressing this FRET probe resulted in FRET changes in a W146-sensitive manner (Fig. 1d), validating this probe. Noticeably, when the cells were stimulated with PDGF instead of S1P, there was a rapid change in the CFP/FRET ratio, which was inhibited in SphK1-siRNA-treated cells (Fig. 1e), strengthening that PDGF utilises S1P_1_ receptor transactivation for maximal chemotaxis. Our previous findings that extracellular α-Syn suppressed PDGF-induced chemotaxis in SH-SY5Y cells^19^ facilitated an investigation of whether α-Syn has any effects on S1P signal. To assess this issue, S1P in place of PDGF was used as an agonist to activate S1P_1_ receptor to simplify the system thereafter.

### α-Syn causes uncoupling of S1P_1_ receptor from Gi

**Figure 2:**
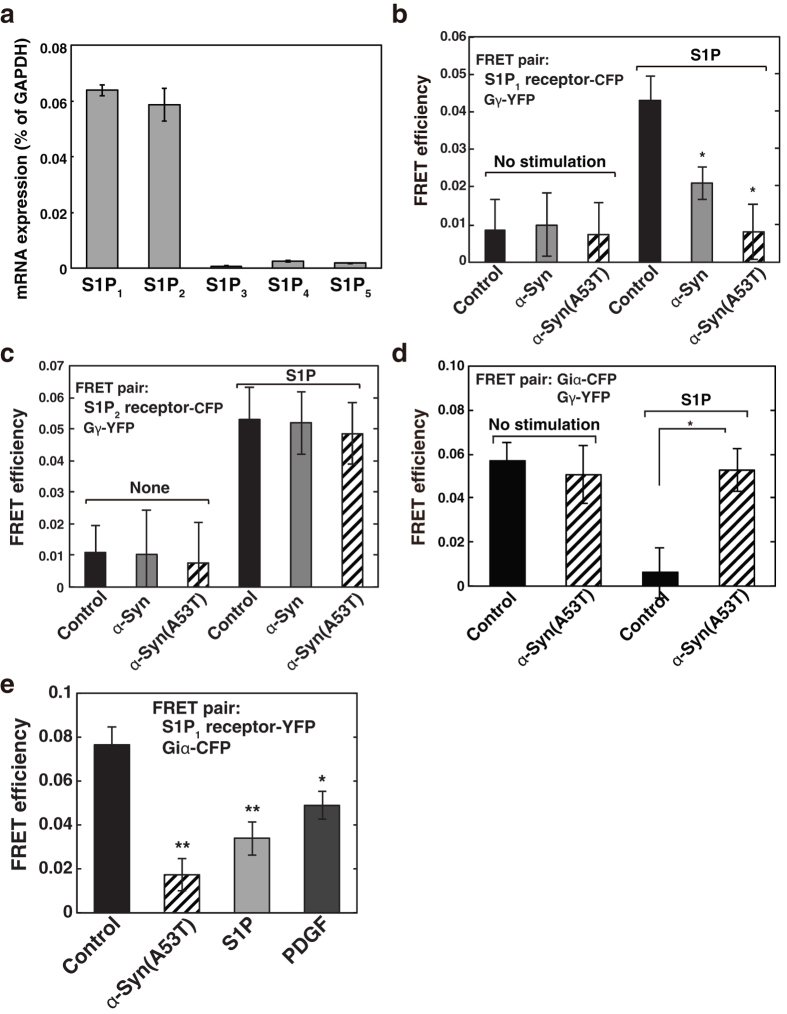
α-Syn-induced uncoupling of S1P_1_ receptor from G-protein, while leaving S1P_2_ receptor unchanged. (**a**) Expression level of S1P_1_, S1P_2_, S1P_3_, S1P_4_ and S1P_5_ receptor mRNAs in SH-SY5Y cells were quantitated by real-time quantitative reverse transcription PCR. Values of mRNA amounts were normalised to GAPDH expression. (**b**) Cells transiently expressing the S1P_1_-CFP, Gβ and Gγ-YFP were pretreated without (closed bars) or with 1 μM wild-type α-Syn (grey bars) or α-Syn(A53T) (hatched bars) for 18 hr and then stimulated with 100 nM S1P for 1 min, fixed and analysed for FRET efficiencies. Values represent means ± s.e.m. (n ≥ 50). Statistical significance was analysed by Student’s t-test (*P < 0.05 versus S1P control). (**c**) SH-SY5Y cells transiently expressing the S1P_2_ receptor-CFP, Gβ and Gγ-YFP were treated with either buffer (control) or 1 μM wild-type α-Syn (grey bars) or α-Syn(A53T) (hatched bars), stimulated with S1P and analysed for FRET efficiencies as in (**b**). Values represent means ± s.e.m. (n ≥ 50). (**d**) Cells transiently expressing the Giα-CFP, Gβ and Gγ-YFP were pretreated without (closed bars) or with 1 μM α-Syn(A53T) (hatched bars) for 18 hr and then stimulated with 100 nM S1P for 1 min, fixed and analysed for FRET efficiencies. Values represent means ± s.e.m. (n ≥ 50). Statistical significance was analysed by Student’s t-test (*P < 0.05). (**e**) Cells transiently expressing the S1P_1_-YFP and Giα-CFP were pretreated without or with 1 μM α-Syn(A53T) for 18 hr and then stimulated with 100 nM S1P or 20 ng/ml PDGF for 1 min, fixed and analysed for FRET efficiencies. Values represent means ± s.e.m. (n ≥ 50; *P < 0.05, **P < 0.01 versus control).

To investigate the effect of extracellular α-Syn on S1P signalling, S1P receptor-mediated downstream events, *i.e.*, G-protein subunit dissociation, were monitored by FRET-based conformational changes. Since SH-SY5Y cells express mainly S1P_1_ and S1P_2_ receptors as judged by real-time quantitative reverse transcription-PCR (Fig. 2a), we focused on these two subtypes of the S1P receptors for S1P signalling. To see the effect of α-Syn on each S1P receptor-mediated signalling, we carried out a FRET analysis using each S1P receptor-CFP and Gγ-YFP as a FRET pair. Under basal conditions heterotrimeric G-protein subunits are associated (Gαβγ form, low FRET). Upon stimulation by S1P, these subunits dissociate, and S1P receptor-CFP and Gγ-YFP become associated (high FRET)^26^. In control cells expressing S1P_1_ receptor-CFP and Gγ-YFP, S1P caused a rapid increase in FRET efficiency, demonstrating a successful detection of S1P_1_ receptor-mediated G-protein subunit dissociation (Fig. 2b). Surprisingly, α-Syn treatment made the receptor refractory toward S1P_1_ receptor-mediated G-protein subunit dissociation. It is notable that α-Syn(A53T), a mutant α-Syn found in familiar PD, caused more potent effects than a wild-type α-Syn with the same concentration (1 μM). On the other hand, when the cells expressing S1P_2_ receptor-CFP and Gγ-YFP were stimulated with S1P, the agonist-induced G-protein dissociation occurred. However, wild-type α-Syn and α-Syn(A53T) treatment showed no significant effects on S1P_2_ receptor-mediated G-protein subunit dissociation (Fig. 2c). These results suggest that α-Syn causes a selective S1P_1_ receptor uncoupling from the G-protein but not S1P_2_ receptor. α-Syn effects were not from cytotoxic ones since S1P_2_ receptor-mediated signalling occurred normally in α-Syn-treated cells (Fig. 2c). Furthermore, two days incubation of the cells with 1 μM α-Syn(A53T) did not cause apoptosis as judged by chromatin condensation assay (Table I). Since the effect of α-Syn(A53T) is clearer than the wild type with the same concentration, subsequent analysis was mainly carried out using α-Syn(A53T).

To demonstrate that α-Syn(A53T) causes S1P_1_ receptor uncoupling from Gi more directly, we have carried out the experiments using another FRET pair with Giα-CFP and Gγ-YFP^26^. Under basal conditions these subunits were associated (high FRET) and S1P_1_ receptor stimulation resulted in the dissociation of the Giα from the Gβγ subunits (low FRET) (Fig. 2d, closed bars). As expected, α-Syn(A53T) treatment abolished the S1P-induced FRET changes (Fig. 2d, hatched bars), indicating that α-Syn(A53T) causes S1P_1_ receptor uncoupling from Gi. Next, to address whether agonist stimulation facilitates S1P_1_ receptor coupling with Gi, the association of S1P_1_ receptor with Giα before and after agonist stimulation was assessed by FRET analysis. Under basal conditions S1P_1_ receptor-YFP was already associated with Giα-CFP as detected by a high FRET efficiency (Fig. 2e, closed bar). S1P and PDGF stimulation caused a significant (light grey bar) and a mild decrease (dark grey bar) in FRET efficiency, respectively. These changes may reflect agonist-induced Gi subunit dissociation. Critically, α-Syn(A53T) caused a significant decrease in FRET efficiency (hatched bar). Taken together with the findings that α-Syn(A53T) impairs the S1P-induced Gi subunit dissociation (Fig. 2d), extracellular α-Syn(A53T) may segregate S1P_1_ receptor from Gi.

### Abrogation of endogenous Gi function by α-Syn(A53T)

**Figure 3:**
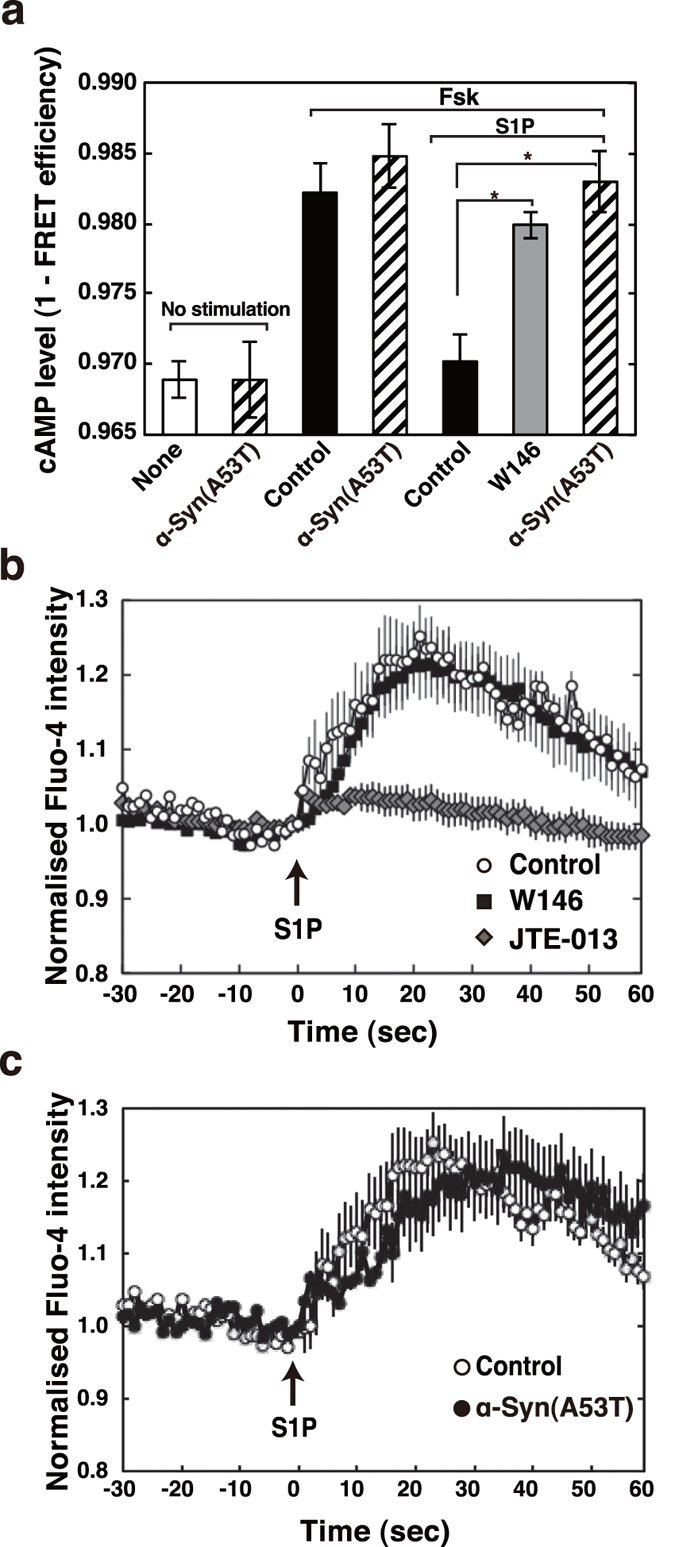
α-Syn-induced impairment of S1P_1_ receptor- but not S1P_2_ receptor-mediated signalling assessed in an endogenous protein system. (**a**) SH-SY5Y cells transiently expressing the cAMP biosensor Epac1-camps were treated with 0.5 mM cAMP phosphodiesterase inhibitor, isobutylmethylxanthine, 20 μM forskolin with or without 100 nM S1P, or 10 μM W146 as indicated. Alternatively, cells transiently expressing the cAMP biosensor, which had been treated with 1 μM α-Syn(A53T) for 18 hr, were stimulated with each agonist as indicated (hatched bars). The FRET efficiency was estimated using acceptor photobleaching. Values represent means ± s.e.m. of 3 independent experiments carried out in triplicate. Statistical significance was analysed by Student’s t-test (*P < 0.05). (**b**) SH-SY5Y cells were serum starved for 18 hr and loaded with 2 μM Fluo-4 AM for 20 min. Cells were washed and pretreated with 10 μM W146 or 10 μM JTE-013 or without (control) for 10 min. The Fluo-4 emission signal for each cell was acquired at a frequency of 1 Hz by fluorescence microscope. After taking basal level signal 100 nM S1P was added (arrow) and the change in fluorescence was monitored. One of the representative quantification results of fluorescence changes in 40 control, 40 W146- and 40 JTE-013-treated cells from 3 independent experiments is shown. (**c**) SH-SY5Y cells were serum starved for 18 hr with or without 1 μM α-Syn(A53T). Cells were then loaded with Fluo-4 AM and stimulated with 100 nM S1P (arrow) in the absence or presence of 1 μM α-Syn(A53T). One of the representative quantification results of fluorescence changes in 40 control and 40 α-Syn(A53T)-treated cells from 3 independent experiments is shown.

The effect of α-Syn(A53T) on endogenous Gi function was assessed next. When cells were treated with S1P, forskolin-stimulated adenylate cyclase activity was potently inhibited (a phenomenon known as a classical Giα function) in a S1P_1_ receptor antagonist W146-sensitive manner (Fig. 3a), evaluating the S1P_1_ receptor/Gi function in an endogenous cell system. Importantly, α-Syn(A53T) abrogated the S1P_1_ receptor/Giα-caused inhibition of forskolin-stimulated adenylate cyclase, supporting the premise that α-Syn(A53T) caused S1P_1_ receptor to uncouple from Gi protein. α-Syn(A53T) itself had no inhibitory effect on forskolin-stimulated adenylate cyclase activity. In contrast, S1P-induced increase in cellular Ca^2+^ through S1P_2_ receptor, which was inhibited by JTE-013 (Fig. 3b), was insensitive to α-Syn(A53T) (Fig. 3c). These results indicate that α-Syn(A53T) causes selective impairment of S1P_1_ receptor/Gi signalling leaving S1P_2_ receptor signalling intact in an endogenous protein system as well as transient expression system (Figs 2 and 3).

### Mechanism underlying α-Syn(A53T)-induced uncoupling of S1P_1_ receptor from Gi

**Figure 4:**
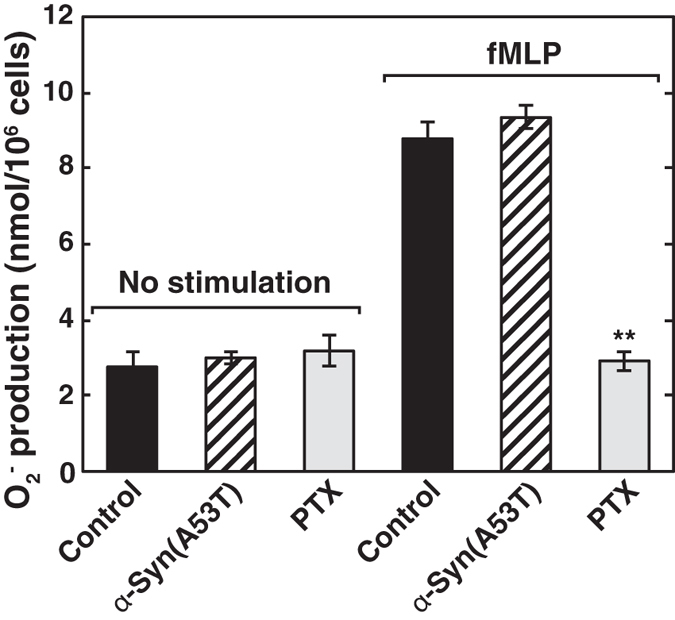
Differential mode of action between α-Syn(A53T) and PTX toward Gi-coupled receptors. Differentiated HL-60 cells were pretreated with 1 μM α-Syn(A53T) or 100 ng/ml PTX for 18 hr. Upon stimulation by 0.1 μM fMLP, superoxide anion production was measured. Values represent means ± s.e.m. of 3 independent experiments carried out in triplicate. Statistical significance was analysed by Student’s t-test (n = 9; **P < 0.01 versus control cells treated with fMLP).

**Figure 5:**
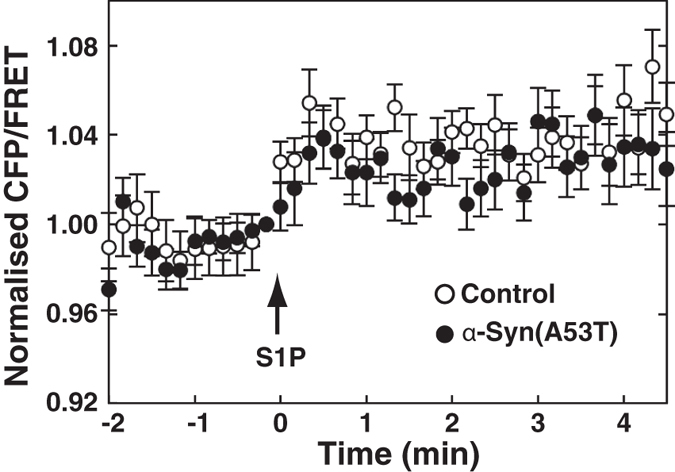
Ineffectiveness of α-Syn(A53T) in S1P-induced conformational changes in S1P_1_ receptor. SH-SY5Y cells transiently expressing the FRET probe as in Fig. 1c were serum-starved for 18 hr in the absence (open circles) or presence (closed circles) of 1 μM α-Syn(A53T). Cells were stimulated with 100 nM S1P (arrow) and analysed for FRET analysis in living cells. A representative emission ratio of the 2 fluorophores from 5 independent experiments is shown.

It is well known that PTX causes G-protein-coupled receptor uncoupling from Gi by ADP-ribosylation of the Giα subunit. The general feature of PTX is to eliminate widely the Gi-dependent phenomena including chemoattractant formyl-Met-Leu-Phe (fMLP)-induced respiratory burst. We therefore undertook a study to compare α-Syn(A53T) with PTX for the ability to cause uncoupling of fMLP receptor. As reported previously^29^, PTX treatment inhibited almost completely fMLP-induced respiratory burst in differentiated HL-60 cells (Fig. 4). In contrast, α-Syn(A53T) had no effects on the phenomena, suggesting that the site of action of α-Syn(A53T) may be distinct from that of PTX. Next, the effect of α-Syn(A53T) on S1P-induced conformational changes in S1P_1_ receptor was assessed using an S1P_1_ receptor FRET tool. S1P-induced conformational changes in the receptor were not influenced by α-Syn(A53T) treatment (Fig. 5, see solid circles versus open circles).

### The effect of α-Syn(A53T) on β-arrestin signal, one of the divergent signals downstream of S1P_1_ receptor

**Figure 6:**
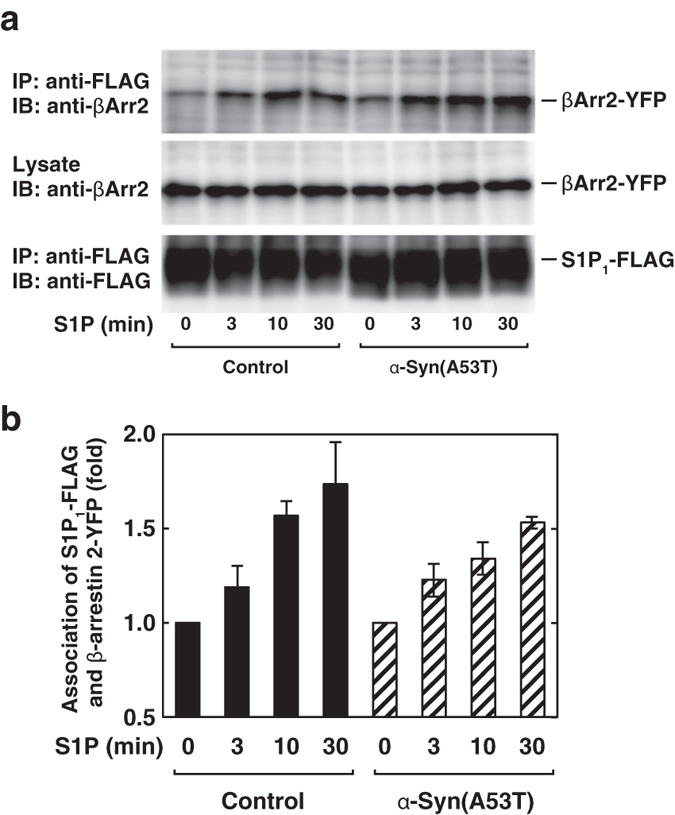
Effect of α-Syn(A53T) on S1P-induced association between S1P_1_ receptor and β-arrestin 2. (**a**) SH-SY5Y cells expressing S1P_1_ receptor-FLAG and YFP-β-arrestin 2 (β-Arr2-YFP) were serum-starved with or without 1 μM α-Syn(A53T) for 18 hr and stimulated by 100 nM S1P for the indicated time periods. Cells were then treated with 2 mM di(N-succinimidyl) 3,3′-Dithiodipropionate (DSP) for 20 min and co-immunoprecipitation assay was done as described in Experimental Procedures. (**b**) The signal intensity of co-immunoprecipitated β-Arr2-YFP in (**a**) was quantified by using Image-J software. Values represent means ± s.e.m. of 3 independent experiments carried out in triplicate.

**Figure 7:**
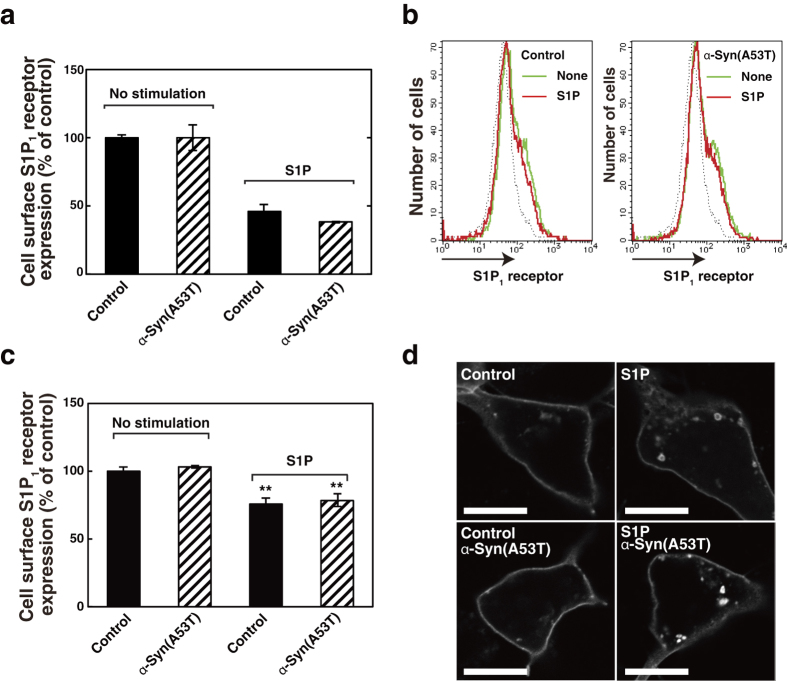
Unaltered response of β-arrestin-mediated signalling after conformational changes in S1P_1_ receptor to α-Syn(A53T). (**a**) S1P_1_ receptor-YFP-expressing SH-SY5Y cells were preincubated at 37 °C with or without 1 μM α-Syn(A53T) for 18 hr in serum-free DMEM/F-12 and treated with or without 100 nM S1P for 1 hr. Cell surface S1P_1_ receptor was then biotinylated, solubilised and pulled-down by streptavidin agarose, followed by measurement of fluorescence of S1P_1_ receptor-YFP. Values represent means ± s.e.m. of 3 independent experiments carried out in triplicate. (**b**) SH-SY5Y cells were treated without or with 1 μM α-Syn(A53T) as in (**a**) and stimulated with 100 nM S1P for 1 hr as specified. Cells were harvested and incubated with anti-S1P_1_ receptor antibody conjugated with Alexa 488, and analysed by flow cytometry. One of the representative histogram plots obtained from three independent experiments is shown. (**c**) Cell surface S1P_1_ receptor was quantitated based on histogram plots in (**b**) and expressed as % of control. Values represent means ± s.e.m. of 3 independent experiments. Statistical significance was analysed by Student’s t-test (**P < 0.01 versus no stimulation). (**d**) S1P_1_-YFP-transfected SH-SY5Y cells were incubated with or without α-Syn(A53T) as in (**a**) and stimulated with 100 nM S1P for 1 hr as specified. Cells were fixed with paraformaldehyde. The localisation of S1P_1_-YFP was analysed by confocal microscopy. Bars, 10 μm. One of the representative results from three independent experiments is shown.

Since G-protein-coupled receptors are known to utilise several other downstream signalling molecules such as β-arrestin as well as G-protein^30^, S1P-induced β-arrestin binding to S1P_1_ receptor was measured. S1P caused association of β-arrestin with S1P_1_ receptor in a time-dependent manner with the maximum level in 30 min (Fig. 6). Importantly, α-Syn(A53T) treatment had little or no effects on the S1P-induced β-arrestin association with the receptor (Fig. 6b, hatched bars). Next, the effect of α-Syn(A53T) on S1P-induced internalisation of the S1P_1_ receptor, one of the well known outcomes of β-arrestin signal^31^, was studied. The cell surface proteins were biotinylated from outside the cells expressing S1P_1_ receptor-YFP before and after S1P stimulation, and then biotinylated S1P_1_ receptor was quantitated. α-Syn(A53T)-treated cells showed a decrease in a cell surface S1P_1_ receptor after S1P stimulation to an extent similar to that in the control cells (Fig. 7a, compare hatched bars with closed bars). This observation was further confirmed by detecting cell surface endogenous S1P_1_ receptor using an antibody to detect extracellular epitope of the receptor. Stimulation of cells with S1P caused a decrease in endogenous cell surface S1P_1_ receptor both in control and α-Syn(A53T)-treated cells to a similar extent (Fig. 7b and c, hatched bars versus closed bars). Furthermore, S1P_1_ receptor internalisation was demonstrated directly in an immunocytochemical study. Upon stimulation of cells with S1P, S1P_1_ receptor-YFP became localised in small punctate structures suggesting S1P_1_ receptor internalisation (Fig. 7d). It should be noted that α-Syn(A53T) had little or no effects on S1P-induced formation of S1P_1_ receptor-positive dot-like structures. Indeed, these results indicate that α-Syn(A53T) causes uncoupling between S1P_1_ receptor and Gi protein, leaving β-arrestin signal unchanged.

## Discussion

Recent studies have revealed that α-Syn can be released from cultured cells by exocytosis^32^ or by exosomes^33^ and that α-Syn is detected in cerebrospinal fluid and plasma^16^,^17^. In addition, cell-to-cell transmission of α-Syn was shown experimentally to induce an inclusion formation and neuronal cell death^34^. From this background we have reasoned that studies on changes in cellular functions induced by exogenous α-Syn may help understand the pathophysiology of α-Syn.

In the present studies we have shown that extracellular α-Syn causes S1P_1_ receptor-selective uncoupling from Gi as determined by both functional (Fig. 3) and FRET-based structural analyses (Fig. 2b,c,d). It has previously been reported that in 1-methyl-4-phenylpyridinium (MPP+) treatment of SH-SY5Y cells, an *in vitro* PD model, there was a significant decrease in SphK1 gene expression and that Sphk1 inhibition plays an important role in caspase-dependent apoptotic neuronal death^35^. However, the mRNA levels of S1P signalling molecules such as SphK1, SphK2, S1P_1_ and S1P_2_ receptors were unchanged under the experimental condition used in the present studies (1 μM α-Syn treatment for 18 hr) although in the proapoptotic conditions such as a higher dose (10 μM) of α-Syn or prolonged time (42 hr) treatment the mRNA level of SphK1 decreased, while that of SphK2 increased (data not shown).

As for the site of action of α-Syn(A53T), it may not act on Gi protein but on S1P_1_ receptor because fMLP-induced respiratory burst was insensitive to α-Syn(A53T) in differentiated HL-60 cells, although it was completely inhibited by PTX, which acts on Giα subunit directly (Fig. 4). α-Syn(A53T) had little or no effect on S1P_2_ receptor as judged by a FRET-based analysis (Fig. 2c) and cellular Ca^2+^ rise in an endogenous protein system (Fig. 3c). Taken together, it may be plausible to assume that α-Syn/α-Syn(A53T) works specifically to S1P_1_ receptor. However, a pull-down of S1P_1_ receptor was not able to detect α-Syn(A53T) association (data not shown). This suggests that α-Syn(A53T) may exert its action indirectly or with the aid of other molecules. In this context it has been shown that the translocation of S1P_1_ receptor to caveolin-enriched microdomains is necessary for the subsequent efficient signalling^36^. In their studies oxidised 1-palmitoyl-2-arachidonoyl-sn-glycero-3-phosphocholine-mediated rapid recruitment to caveolin-enriched microdomains of signalling molecules including the S1P_1_ receptor and Akt is important in endothelial barrier enhancement in human pulmonary endothelial cells. It has been suggested that α-Syn has an ability to interact with gangliosides in the cholesterol and sphingolipid-rich membrane microdomains known as lipid rafts, and has a potency to alter the functions of several signalling molecules at the raft regions^15^. In these lines it has been shown that disruption of lipid raft by methyl-β-cyclodextrin caused impairment of G-protein effector signalling but not α1a-adrenergic receptor internalisation^37^. To support this we have recently observed that ganglioside binding-deficient mutant of α-Syn(A53T), α-Syn(A53T)-AAA, lost its ability to suppress PDGF-induced chemotaxis in SH-SY5Y cells^19^. Consequently, it may be possible that α-Syn/α-Syn(A53T) causes changes in the membrane microdomain environment, which in turn alters S1P_1_ receptor function.

The present results also show that another downstream signalling of S1P_1_ receptor, β-arrestin-involved signalling was insensitive to α-Syn(A53T) (Fig. 6) and its physiological effect-S1P-induced internalisation of the S1P_1_ receptor was unaffected by α-Syn(A53T) (Fig. 7). It has been shown that β-arrestin binding requires the ligand-induced conformational changes of the G-protein-coupled receptor and the subsequent receptor phosphorylation^38^. We have shown that α-Syn(A53T) caused no effects on the S1P-induced conformational changes in the S1P_1_ receptor as judged by FRET-based studies (Fig. 5). These conformational changes may trigger phosphorylation of the receptor necessary for subsequent β-arrestin binding. The demonstration here indicates that exogenous α-Syn modulates S1P_1_ receptor-mediated signalling from both Gi and β-arrestin signals into β-arrestin-biased one by uncoupling of the receptor from Gi.

Although the discovery of hereditary forms of PD has contributed greatly to understand the pathogenesis of the disease, that of sporadic PD, the majority forms of PD, is still elusive. It has recently been revealed that mutations in the GBA and SMPD1 genes are risk factors for PD. GBA encodes the lysosomal enzyme glucocerebrosidase that catalyses the breakdown of the glycolipid glucosylceramide to ceramide and glucose. GBA mutations, when homozygous, lead to Gaucher’s disease, while predispose to PD when heterozygous^39^. SMPD1 mutations that cause Niemann-Pick type A were significantly higher in patients with PD compared to young controls^40^. SMPD1 encodes sphingomyelin phosphodiesterase 1 (acid sphingomyelinase), a lysosomal enzyme that hydrolyzes sphingomyelin to generate phosphorylcholine and ceramide. Interestingly, both gene products share a common feature, *i.e.*, their enzymatic products are ceramide. Ceramide is shown to be involved in exosomal vesicle formation in multivesicular endosomes (MVEs)^41^. Furthermore, we have recently reported that continuous activation of S1P_1_ receptor by S1P, a further metabolite of ceramide, on MVEs has an essential role in the cargo sorting into exosomes^26^. Along with the evidence that α-Syn is secreted in the form of exosomes^33^, it is tempting to speculate that uncoupling of S1P_1_ receptor from Gi caused by extracellular α-Syn may inhibit exosomal release of α-Syn, which results in the accumulation of α-Syn inside the cells, a pathological hallmark of PD. Further studies on the mechanism underlying extracellular α-Syn-caused uncoupling of S1P_1_ receptor from Gi will be the keys to understand the pathogenesis of sporadic PD.

## Methods

### Reagents

S1P was purchased from Enzo Life Sciences; PTX, forskolin and dibutyryl cAMP from Wako Pure Chemical Industries; fMLP and cytochrome c were from Sigma Aldrich. W146 and JTE-013 were from Cayman Chemical Company. Other reagents and chemicals were of analytical grade.

### Plasmids and mutations

Human α-Syn was amplified and subcloned into the bacterial expression vector pET3a. For α-Syn(A53T), alanine 53 was mutated to a threonine using a QuikChange site-directed mutagenesis protocol. mS1P_1_ receptor-CFP, Giα-CFP, Gβ and Gγ-YFP plasmid constructs were prepared as described previously^26^. Murine S1P_2_ (mS1P_2_) receptor (GenBank accession number NM_010333.4) cDNA was amplified from mouse brain cDNA, which had been reverse transcribed from fetal mouse brain mRNA (Invitrogen) by PCR (sense primer, 5′-CGGAATTCGCCACCATGGGCGGCTTATACTCAGAG-3′; antisense primer, 5′-CGGAATTCGGACCACTGTGTTACCCTCCAG-3′) to make a C-terminally CFP-fused construct in pECFP-N1. A one-molecular FRET probe for detection of cAMP, Epac1-camps, was constructed as reported previously^42^. For one-molecule FRET probe for the detection of conformational changes of S1P_1_ receptor, EYFP was inserted in the third intracellular loop of murine S1P_1_ receptor between Lys243 and Ala244. Briefly, the cDNA encoding Met1 to Lys243, Ala244 to Ser382 of mS1P_1_, or EYFP was amplified by PCR using 5′-CGGGAATTCGCCACCATGGTGTCCACTAGCATCC-3′ and 5′-ATAGGATCCCTTGGAGATGTTCTTGCG-3′, 5′-GCTCTAGAGCCAGTCGCAGTTCTGAG-3′ and 5′-CGGAATTCGGGAAGAAGAATTGACGTTTCCAG-3′, or 5′-ATAGGATCCATGGTGAGCAAGGGCGAG-3′ and 5′-GCTCTAGACTTGTACAGCTCGTCCATG-3′, respectively. Each cDNA fragment was treated with EcoRI and BamHI, XbaI and EcoRI or BamHI and XbaI, respectively, and these three fragments were inserted into EcoRI-cut pECFP-N1. All the constructs were verified by sequencing.

### siRNA

For RNA interference following oligonucleotides (Japan Bio Services, Saitama, Japan) were used: Sense 5′-GGAGAACAGCAUUAAACUGdTdT-3′ and antisense 5′-CAGUUUAAUGCUGUUCUCCdTdT-3′ for human S1P_1_ receptor;sense 5′-GGGCAAGGCCUUGCAGCUCdTdT-3′ and antisense 5′-GAGCUGCAAGGCCUUGCCCdTdT-3′ for human SphK1;sense 5′-UUCUCCGAACGUGUCACGUdTdT-3′ and antisense 5′-ACGUGACACGUUCGGAGAAdTdT-3′ for control.

SH-SY5Y cells were transfected with the siRNAs using Lipofectamine RNAiMAX according to the manufacturer’s instructions (Invitrogen, Carlsbad, CA, USA).

### Bacterial expression and purification of recombinant α-Syn and α-Syn(A53T)

Recombinant α-Syn and α-Syn(A53T) were expressed in E. Coli and purified as described previously^43^. Briefly, α-Syn or α-Syn(A53T) cDNAs subcloned into pET3a was transformed in E. coli BL21 (DE3) and protein expression was induced by 0.1 mM IPTG for 3 hr. Bacterial pellets were resuspended in TE buffer (10 mM Tris-HCl, pH7.5 and 1 mM EDTA) containing 750 mM NaCl (TE-750 mM NaCl) with protease inhibitors, heated at 100 °C for 10 min, and centrifuged at 70,000 × g for 30 min. The supernatant was dialysed against TE-20 mM NaCl, filtered by 0.22 μm filter and applied to a Mono S column (GE Healthcare). The unbound fractions were applied to a Mono Q column (GE Healthcare). α-Syn was eluted with a 0–0.5 M NaCl linear gradient. The fractions containing α-Syn were identified by Coomassie Brilliant Blue staining and immunoblot analysis following SDS-PAGE. Protein concentration was determined using Bradford protein assay kit (Bio-Rad).

### Cell cultures and transfections

SH-SY5Y cells (American Type Culture Collection, CRL-2266) were maintained in DMEM/F-12 medium (Wako Pure Chemical Industries) containing 10% fetal bovine serum and 1% penicillin/streptomycin at 37 °C in 5% CO_2_. HL-60 cells were grown in RPMI 1640 medium (Wako Pure Chemical Industries) containing 10% fetal bovine serum and 1% penicillin/streptomycin at 37 °C in 5% CO_2_. Cells were plated onto 35 mm glass-bottom culture dishes (MatTek) before transfection. Transient transfection was carried out using FuGENE HD (Promega). All experiments were performed 2 to 3 days after transfection.

### S1P_1_ receptor internalisation assay

SH-SY5Y cells expressing S1P_1_ receptor-YFP were incubated at 37 °C with or without 1 μM α-Syn(A53T) for 18 hr in serum-free DMEM/F-12 and treated with or without 100 nM S1P for 1 hr. Cells were then treated with sulfo-NHS-SS-biotin (Thermofisher Scientific) at 4 °C for 30 min and solubilised with lysis buffer (20 mM Tris-HCl, pH 7.4, 150 mM NaCl, 1 mM EDTA, 1% Triton X-100 and protease inhibitors), followed by centrifugation at 10,000 × g for 15 min at 37 °C. Biotinylated S1P_1_ receptor-YFP was pulled-down by streptavidin agarose beads (Solulink Biosciences, Inc.) for 1 hr at 4 °C and the amount of biotinylated cell-surface S1P_1_ receptor-YFP was directly measured using an EnSpire plate reader (Perkin-Elmeer). Values represent means ± s.e.m. of 3 independent experiments carried out in triplicate. To detect endogenous S1P_1_ receptor internalisation, SH-SY5Y cells (1 × 10^6^ cells) were serum-starved in the absence or presence of 1 μM α-Syn(A53T) for 18 hr. After cell stimulation with S1P, cells were chilled on ice and harvested by using Cell Dissociation Buffer (Life Technologies, Inc.). Cells were washed with PBS containing 1% sodium azide and 1% BSA and incubated with 1 μg/ml of anti-S1P_1_ receptor antibody conjugated with Alexa 488 (Novus Biologicals), which detects endogenous cell surface S1P_1_ receptor from outside of the cells, at 4 °C for 1 hr, followed by washing and analysed by flow cytometry (FACScalibur, BD Biosciences).

### Real-time quantitative reverse transcription-PCR

Total RNA was extracted from SH-SY5Y cells (2 × 10^6^ cells) using NucleoSpin RNA II (Macherey-Nagel) according to the manufacturer’s instructions. 1 μg RNA was used for reverse transcription (ReverTra Ace qPCR RT kit, TOYOBO). Quantitative PCR was performed with SYBR Premix (Takara) on ABI Prism 7000. Expression level of glyceraldehyde 3-phosphate dehydrogenase (GAPDH) was used as internal control for normalisation. The primer sequences (sense and antisense) were as follows: for human S1P_1_ receptor, 5′-TTCCTGGTGTTAGCTGTGCTCAAC-3′ and 5′-TCGCTTGAATTTGCCAGCAGAGTC-3′; for human S1P_2_ receptor, 5′-TGCGCCATTGTGGTGGAAAACC-3′ and 5′-TTGCCCAGAAACAGGTACATTGCC-3′; for human S1P_3_ receptor, 5′-AGCAGCAACAATAGCAGCCACTC-3′ and 5′-AGTGCTGCGTTCTTGTCCATGATG-3′; for human S1P_4_ receptor, 5′-AACTGCCTGTGCGCCTTTGAC-3′ and 5′-ATCACCAGGCAGAAGAGGATGTAGC-3′; for human S1P_5_ receptor, 5′-TTCCTGCTGCTGTTGCTCGAC-3′ and 5′-TTCAGAAGTGAGTTGGCCATGGC-3′; for human GAPDH, 5′-GCCATCAATGACCCCTTCATT-3′ and 5′-TCTCGCTCCTGGAAGATGG-3′.

### Acceptor photobleaching

SH-SY5Y cells were transiently cotransfected with S1P_1_-CFP or S1P_2_-CFP, Gβ and Gγ-YFP^26^, with a donor/acceptor ratio of 1:1:1, with one-molecule cAMP probe, Epac1-camps^42^, with Giα-CFP, Gβ and Gγ-YFP^26^ or Giα-CFP and S1P_1_-YFP. Two days after transfection, cells were serum-starved in the absence or presence of 1 μM α-Syn or α-Syn(A53T) for 18 hr and treated with various reagents. Cells were then fixed and each area of interest was subjected to FRET analysis with acceptor photobleaching method using a LSM 510 META with a 63 x oil plan-apochromat objective. Following excitation at 458 or 514 nm, CFP emission with a 475–525-nm band-pass barrier filter or YFP emission with 530–600-nm band-pass barrier filter, respectively, was collected. An area of interest was selected for photobleaching of YFP. An automated acquisition protocol was then used, which recorded pre- and post-bleaching images using 458 nm excitation at 8% laser power to limit photobleaching, with a bleaching of the selected area with 100%, 514 nm laser power with 50 iterations (acceptor photobleaching). FRET was resolved as an increase in the CFP (donor) signal after photobleaching of YFP (acceptor). FRET efficiency (E) can be determined from the relative fluorescence intensity of the energy donor (CFP) before (Ipre) and after (Ipost) photobleaching of the energy acceptor (YFP): E = 1 − (Ipre/Ipost).

### Measurement of superoxide anion production in differentiated HL-60 cells

HL-60 cells were cultured for 48 hr with 0.2 mM dibutyryl cAMP to induce differentiation followed by serum starvation in the absence or presence of 1 μM α-Syn(A53T) or 100 ng/ml PTX for 18 hr. Differentiated HL-60 cells were suspended in the HEPES-buffered medium consisting of 10 mM HEPES/NaOH (pH 7.4), 130 mM NaCl, 4.7 mM KCl, 1 mM CaCl_2_, 1.2 mM KH_2_PO_4_, 1.2 mM MgSO_4_, 10 mM glucose and 0.2% BSA with or without 1 μM α-Syn(A53T). Cell suspension (10^6^ cells/tube) was treated with 0.1 μM fMLP and 50 μM cytochrome c at 37 °C for 10 min followed by rapid centrifugation. The cell superoxide anion production was estimated by measuring the reduction of cytochrome c as the increase in absorbance at 550 nm using a spectrophotometer.

### Ca^2+^ measurements

SH-SY5Y cells seeded on glass bottom dish were incubated with or without 1 μM α-Syn(A53T) for 18 hr in serum-free DMEM/F-12. Cells were loaded with 2 μM Fluo-4 AM (Dojindo Laboratories) in HEPES-buffered medium consisting of 10 mM HEPES/NaOH (pH 7.4), 130 mM NaCl, 4.7 mM KCl, 1 mM CaCl_2_, 1.2 mM KH_2_PO_4_, 1.2 mM MgSO_4_, 10 mM glucose with or without 1 μM α-Syn(A53T) at 37 °C for 20 min and washed. Cells were pretreated with 10 μM W146 or 10 μM JTE-013 or without (control) for 10 min. In some experiments cells were treated with 1 μM α-Syn(A53T) for 10 min. The glass bottom dish was mounted on IX70 fluorescence microscope (Olympus) and Fluo-4 fluorescence was recorded. Excitation wavelength was set to 490 nm and emission was recorded at 535 nm. After taking basal level fluorescence for 30 s, 100 nM S1P was added and the change of fluorescence was monitored at a frequency of 1 Hz. The acquired Fluo-4 emission signal for each cell was normalised to the basal signal and plotted against time.

### Co-immunoprecipitation assay between S1P_1_ receptor-FLAG and β-arrestin 2-YFP

SH-SY5Y cells expressing S1P_1_ receptor-FLAG and β-arrestin 2-YFP were serum-starved for 18 hr with or without 1 μM α-Syn(A53T) and stimulated with 100 nM S1P for the indicated time periods. Cells were then treated with 2 mM Di(N-succinimidyl) 3,3′-Dithiodipropionate (DSP) (Tokyo Chemical Industry Co., LTD.) for 20 min at room temperature, followed by solubilisation with lysis buffer (40 mM Tris-HCl, pH 7.4, 100 mM NaCl, 1 mM EDTA, 10 mM NaF, 0.2% n-dodecyl β-D-maltoside (DDM) and protease inhibitor cocktail (Nacalai tesque)). Cell lysates were cleared by centrifugation at 13,000 × g for 15 min and immunoprecipitated by anti-FLAG beads (Wako Pure Chemical Industries). The immunoprecipitates were separated by SDS-PAGE and co-immunoprecipitated β-arrestin 2-YFP was detected by rabbit monoclonal anti-β-arrestin 2 antibody (Cell Signaling Technology) in immunoblotting.

### Apoptosis assay

SH-SY5Y cells were serum-starved in the absence or presence of 1 μM wild-type α-Syn or α-Syn (A53T) for the indicated time periods. Cells were stained with 4,6-diamidino-2-phenylindole-2-HCl (DAPI) and chromatin condensation during apoptosis was visualised by using LSM510 confocal microscope.

### Statistical analysis

Results are expressed as means ± s.e.m. Data were analysed by t-test. P-values < 0.05 were considered significant.

## Additional Information

**How to cite this article:** Zhang, L. *et al*. Extracellular α-synuclein induces sphingosine 1-phosphate receptor subtype 1 uncoupled from inhibitory G-protein leaving β-arrestin signal intact. *Sci. Rep.* **7**, 44248; doi: 10.1038/srep44248 (2017).

**Publisher's note:** Springer Nature remains neutral with regard to jurisdictional claims in published maps and institutional affiliations.

## Figures and Tables

**Table 1:** SH-SY5Y cells were serum-starved in the absence or presence of 1 μM wild-type α-Syn or α-Syn(A53T) for indicated time periods.

Conditions	Apoptotic cells	
	18 hr%	48 hr%
Serum-starved	1.47 ± 0.24	3.36 ± 0.33
α-Syn (serum-starved)	1.18 ± 0.45	3.09 ± 0.57
α-Syn(A53T) (serum-starved)	1.17 ± 0.46	3.02 ± 0.22

Chromatin condensation during apoptosis was detected by 4,6-diamidino-2-phenylindole-2-HCl (DAPI) staining. Data are mean ± s.e.m. of 3 independent experiments. No significant difference was seen between the treatments.
