# Erratum: Extracellular α-synuclein induces sphingosine 1-phosphate receptor subtype 1 uncoupled from inhibitory G-protein leaving β-arrestin signal intact

**DOI:** 10.1038/srep46964

**Published:** 2018-04-06

**Authors:** Lifang Zhang, Taro Okada, Shaymaa Mohamed Mohamed Badawy, Chihoko Hirai, Taketoshi Kajimoto, Shun-ichi Nakamura

Scientific Reports 7: Article number: 44248; 10.1038/srep44248 published online: 03 16 2017; updated: 04 06 2018.

The original version of this Article contained an error in the indexing of the author Shaymaa Mohamed Mohamed Badawy. This error has now been corrected.

